# Evaluation of Oral and Dental Health Results and Competition Stress Levels of Adolescent Athletes in Different Winter Sports Branches

**DOI:** 10.5152/eurasianjmed.2024.23281

**Published:** 2024-06-01

**Authors:** Buket Sevindik Aktaş, Fatih Şengül, Fatih Kıyıcı

**Affiliations:** 1 Department of Coaching Education, Erzurum Technical University Faculty of Sport Sciences, Erzurum, Türkiye; 2 Department of Pediatric Dentistry, Atatürk University Faculty of Dentistry, Erzurum, Türkiye; 3 Department of Physical Education and Sport, Atatürk University Faculty of Sport Sciences, Erzurum, Türkiye

**Keywords:** Bruxism, cortisol, dental trauma, oral health, stress, winter sports

## Abstract

Background: The aim of the present study was to examine the findings of the intraoral examinations of the 12-16 years old novice (control) and elite athletes across the winter sports branches, which were alpine discipline skiing, snowboarding, biathlon, ski jumping, and ice hockey. Besides, the study aimed to determine the intra- and inter-group relationships by comparing the athletes salivary stress biomarker levels at pre-competition, during-match, and post-competition stages.

Methods: Ninety-one athletes (71 elite, 20 novice) participated in our study. Oral health status of the athletes were evaluated. In addition, cortisol levels in the saliva samples obtained at pre-competition (rested before the competition), during-match (just before the start), and post-competition (competition ending moment) stages were measured. The data were analyzed statistically with a significance level of 0.05.

Results: Ice hockey athletes were the most affected by untreated dental caries (27.3%). No signs of dental trauma caused by sports activities were found in the winter sports branches. Basic erosive wear examination scores of the ice hockey athletes were similar to the ski jumping athletes and lower than other groups (P = .034). The mean cortisol values between sports branches were listed as: biathlon > snowboarding > alpine skiing > ice hockey > ski jumping (P < .001). Test results of the winter sports athletes’ saliva samples revealed that their salivary stress levels might vary in accordance with the sports branches, and there was a negative relationship between the levels of salivary stress biomarkers in competitions and oral health.

Conclusion: In winter sports activities, measures for improving oral health should be disseminated.

**Main Points**

- Among all sports branches, the lowest dental attrition scores seen in the ice hockey players are supporting the efficacy of the mouth guards.
- Post-competition salivary stress biomarker levels of the athletes were found to be higher than the pre-competition and during-match stress levels.
- Cortisol levels, measured in saliva, may promote the optimization of athletes’ training and match standards.

## Introduction

Sports activities that improve an individual’s quality of life, self-confidence, and friendships are affected by oral and dental health.^1 2^ As a part of physical health, the presence of an oral health problem adversely affects the athlets’ performances in the short term, and also their health is negatively impacted in the long term.^3-5^ Needleman et al^6^ reported that professional athletes had poor oral hygiene. Vanhegan et al^7^ also stated that 30% of the athletes participating in the increase applied to their dentists due to dental problems.^7^

Various indices are used in studies on the oral/dental health of the individuals who do and do not play sports.^8,9^ The number of decayed, missing, and filled teeth in individuals is determined by the Decayed, Missing, and Filled Teeth (DMFT) index. The Decayed, Missing, and Filled Surfaces (DMFS) index indicates the number of affected tooth surfaces in these teeth.^10^ The Pulpal involvement, ulceration, fistula, and abscess (PUFA) index is used to evaluate the oral conditions resulting from untreated caries.^11^ Simplified oral hygiene index (OHI-S) score is used for assessing the oral hygiene status of individuals (good: 0-1.2, fair: 1.3-3.0, and poor: 3.1-6.0) based on 6 tooth surfaces.^12^ Basic erosive wear examination (BEWE) index is used to determine the amount of tooth erosion.^5^ In addition, the level of the cortisol hormone in the blood, saliva, and gingival crevicular fluid can also be determined to measure the stress level of the individuals.^13,14^ Cortisol determination from saliva is preferred as it is a non-anxious, non-invasive, and easy-to-apply method compared to blood collection with a syringe.^10^

Winter sports are divided into 2 main branches, which are played on snow or ice.^15^ There are branches such as alpine skiing, nordic skiing, snowboarding, and biathlon in the snow; and ice hockey in the ice sports. Alpine skiing is a competition with skiing that covers characteristics such characteristics as technique, courage, speed, risk, fitness, and determination. It also contains a lot of adrenaline.^16^ Nordic discipline, which requires a high level of aerobic endurance, covers the types of gliding in the ski running branch. In order to move more easily on the snow, the feet are released from the heels and are connected to the skis only from the toes.^17^ Ski jumping is another branch of the Nordic discipline in which competitors aim to jump the furthest distance with their aerodynamic structures by sliding on their skis from specially designed ramps.^17^ In the field of snowboarding, where 2 legs are fixed on 1 board and moved in the lateral direction, athletes’ characteristics such as technique, courage, speed, determination, and conditioning stand out.^1^ Biathlon is a stressful and popular sport in which skiing and rifle marksmanship are combined, and it is recognized as 1 of the 5 most difficult sports in the world.^18^ Ice hockey, which has a hard and fast nature, is among the fastest sports played on ice, and the competitions are held with 2 teams.^19^

There is no information in the literature regarding whether there is a relationship between the oral findings of the athletes across the winter sports branches and their stress levels during the competitions. Thus, we aimed to assess the oral and dental health status of the elite athletes nationwide representing Türkiye in international competitions in alpine skiing, snowboarding, biathlon, ski jumping, and ice hockey. Furthermore, we compared their intra- and inter-sports branch salivary stress levels.

## Material and Methods

As given in the acknowlegement ethics section for this study, Türkiye’s Official permissions were obtained from Atatürk University Winter Sports and Sports Sciences Institute Ethics Committee (decision dated 06.08.2018 and numbered E.1800230002), the Presidency of Turkish Ski Federation (decision dated 01.11.2018 and numbered E.1800315924), the Ice Hockey Federation Presidency (decision dated 01.11.2018 and numbered 2349) and the Turkish Ice Skating Federation Presidency (dated 13.11.2018 and numbered E.1800327691). Since all participating athletes were under 18 years of age, written informed consent forms were collected from both the athletes and their families. All procedures were performed in accordance with the ethical standards of the Helsinki Declaration.

In Türkiye, since the 25th Universiade Winter Games held in 2011, international winter competitions have all been hosted in Erzurum, a city at an elevation of 3200 meters (10 498 feet) where it became a center for winter sports due to its mountainous terrain and cold climate. Present study included all nationwide elite athletes aged 12-16 years representing Türkiye in the international competitions in alpine skiing, snowboarding, biathlon, ski jumping, and ice hockey. The study consisted of a total of 71 athletes with no health problems and a control group of 20 individuals of similar age who do not participate in any sports activities and have no health issues.

### Intraoral Examination

Data had been collected during 2019 by 1 experienced pediatric dentist (E.K.). Calibration was conducted by comparing the previous oral examination results of the 15 selected athletes (examined by E.K.) to the diagnoses remade by 1 of the authors (F.S.) until the level of agreement had reached 90%. Examinations were conducted in our dental clinics, employing a dental chair and an operating light. Teeth were air-dried, and a plain dental mirror was used.

In the oral examinations, DMFT, DMFS, PUFA scores, OHI-S values, BEWE scores, presence of bad oral habits, and dental trauma were evaluated and recorded in the examination forms.

DMFT and DMFS measurements of the athlete and control groups were made in accordance with World Health Organization criteria.^20^ Visible caries lesions on all tooth surfaces were recorded. DMFT index for each individual’s permanent teeth is calculated by counting D (number of decayed teeth), M (number of missing teeth), and F (number of filled teeth). When calculating this index, the number of decayed, missing, filled teeth is divided by the number of people examined. Thus, decayed, missing, and filled teeth per person are calculated (D+M+F/ N = DMFT). Unlike the DMFT index, in the DMFS index, calculations are based on the affected tooth surfaces. The DMFS measures the severity of dental caries according to the surface. In the DMFS index, anterior teeth and molars are noted as 4 and 5 surfaces, respectively.

In our study, PUFA index scores were developed by Monse et al^11^ in 2010 and evaluating the oral findings associated with untreated dental caries were calculated. PUFA is an index used to assess the presence of oral conditions resulting from untreated caries. The codes of the PUFA index are recorded according to the presence of dental caries involving the pulp, oral ulcerative lesions stimulated by retained root fragments, fistula, and abscess.^11^

In the present study, OHI-S scoring was used to determine the oral hygiene score. Oral hygiene status depending on the OHI-S score was classified as good (0-1.2), moderate (1.3-3), and poor (3.1-6) and recorded in the clinical examination forms.

In order to determine the amount of tooth erosion in the participants, the BEWE was used.^21^ The buccal, occlusal, and lingual/palatal surfaces of the teeth, which are divided into 6 regions in the mouth, are graded between 0 and 3 in terms of erosion severity and they are the lowest in each region. The total BEWE score was calculated by summing the high scores.

Individuals were questioned and examined for bad oral habits such as bruxism, teeth grinding, lip biting, cheek biting, and pencil biting. Also, the clinical findings of dental trauma types were evaluated and recorded as enamel fracture, enamel-dentin fracture, complex enamel-dentin fracture, crown-root fracture, subluxation, lateral luxation, intrusion extrusion, and avulsion in the examination form.

### Saliva Collection Procedure and Evaluation

Participants were asked not to take any nutrients or drugs, not to use cosmetics such as lipstick an hour before their unstimulated saliva samples were taken, and they were informed to perform oral care the night before.

For each measurement, saliva samples of the users were collected in different centrifuge tubes (Isolab, Wertheim, Germany). Saliva samples of the participants in the athlete group were collected at pre-competition (rested before the competition), during-match (just before the start), and post-competition (competition ending moment) stages, and saliva samples of the control group were obtained only at their rest time.

The unstimulated saliva samples of the individuals were collected between 9:00 am and 12:00 pm, while the individuals were in a resting position, using the “spitting method” in which the subjects spit into the tubes given. For cortisol analysis of the saliva samples, each tube was sealed and sent to the laboratory in an icebox, and the collected samples were stored at −23°C until the time of analysis.

Cortisol level of saliva samples was determined by Human Cortisol ELISA (enzyme-linked immunosorbent assay) kit (YLA0169HU, YL Biotech Co. Ltd., Shanghai). The salivary cortisol hormone measurements were made in the ELISA device at 450 nm and the results were recorded, and they were calculated as ng/mL by drawing the absorbance–concentration curve.

### Statistical Analysis

The study data were analyzed using the SPSS 20 (IBM SPSS Corp.; Armonk, NY, USA). Since our data are nonparametric, age, DMFT, DMFS, OHI-s, BEWE, pre-competition, DM, and post-competition values were analyzed using Mann–Whitney, Kruskal–Wallis, and Friedman tests and in the presence of trauma, the chi-square test was used. The level of significance was accepted as *P* < .05.

## Results

The present study includes a total of 91 people between the ages of 12-16, 71 of whom are the competitors (elite) in their sport branches and 20 of them are in the control group. The average age of the athletes participating in the study is given in Table 1. The mean age of snowboard and ski jumping branches was lower than the control group (*P* < .001).

DMFT, DMFS, PUFA, and OHI-S values of athletes in the branches are shown in Table 2. The mean DMFT and DMFS scores were found to be the lowest in the control group and the highest in the alpine branch. However, no statistically significant difference was found between the sports branches in DMFT and DMFS values (*P*_DMFT _= .083, *P*_DMFS _= .077). No PUFA findings were observed in the ski jumping and biathlon branches, while the highest prevalence of PUFA was found in the ice hockey branch (n = 6, 27.3%). Athletes were found to have worse oral hygiene than the control group. In addition, the mean OHI-S score of the biathlon group with the worst oral hygiene was found to be statistically higher than the snowboard group (*P* = .018).

**Figure 1.**
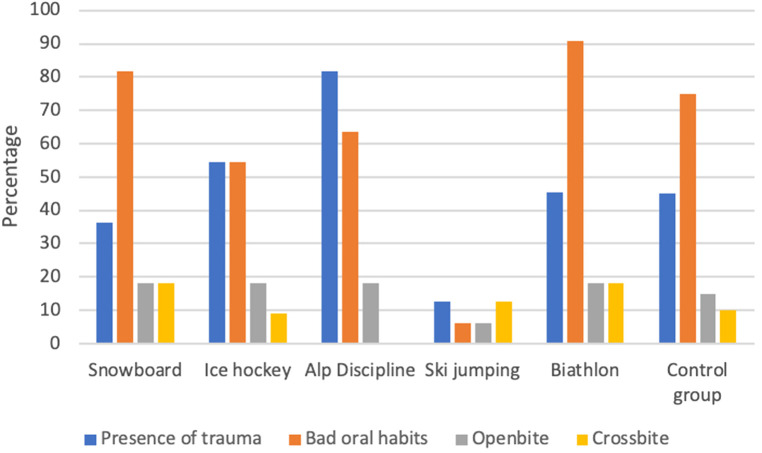
Distribution of the percentages of oral trauma and bad habits observed in athletes.

The distribution of intraoral findings of athletes competing in the branches is given in Figure 1. In the intraoral examinations, signs of dental trauma were found in 45.1% of the athletes. The presence of trauma was the lowest in the ski jumping branch (12.5%) and the highest in the alpine discipline branch (54.5%) (*P* = .86). Among the types of dental trauma detected in athletes, enamel fracture (41.8%) was the most common and accompanied by a few enamel-dentin fractures (2.2%) and subluxation (1.1%). In the athletes’ anamnesis, it was revealed that the traumas did not occur during the trainings or competitions.

In the dental examination, bad oral habits including bruxism, teeth grinding, lip biting, cheek biting, and pencil biting were found in 59.3% of the athletes (Figure 1). The rate of bad oral habits was found to be the highest (90.9%) in biathlon and the lowest (6.3%) in ski jumping (*P* = .86). While 15.4% of the athletes had an open bite, 11.0% had a crossbite. The remaining 73.6% of the athletes did not have any occlusal problems.

The BEWE scores indicating the wear on the teeth of the athletes across the different branches are given in Table 3. The BEWE score of the athletes in the ice hockey branch was found to be similar to the ski jumping branch but lower than the other groups (*P* = .034).

In order to determine the stress levels of the athletes, their levels of salivary cortisol measured at pre-competition, during-match, and post-competition stages are given in Table 4. In the biathlon branch, significantly higher cortisol levels were detected than in other branches at all 3 times of measurement (*P* < .001). Cortisol levels of the alpine skiing and ski jumping groups before the competitions were significantly lower than the control group (*P* < .001). It has been observed that the ski jumping branch, which had the lowest cortisol level at during-match stage, had a similar cortisol level with only the ice hockey (*P* < .001). It was determined that the ski jump branch had a significantly lower cortisol level than other branches at post-competition stage (*P* < .001).

Mean salivary cortisol levels of professional athletes measured at the post-competition stage were observed to be statistically higher than the other measurement stages (*P* < .05). There was no significant difference between cortisol levels measured at 3 different stages in the sports branches except for alpine skiing (*P* > .05). The post-competition cortisol levels in the Alpine skiing branch were statistically higher than pre-competition levels (*P* = .029).

## Discussion

The positive effects of sporting activities on the growth and development of children have been expressed in scientific studies.^22,23^ Studies examining how sporting activities are related to growth and development have mostly focused on physical development, and there is a scarcity of research on its association with oral and dental health.^24,25^ These studies are mostly related to dental caries, erosions, and traumatic injuries occurred in the individuals engaged in sports, yet limited studies have explored the relationship between winter sports athletes, cortisol, and oral and dental health.^8,26,27^ The review of the literature revealed no study investigating the effects of stress caused by competitions in winter sports disciplines on salivary stress hormones and oral and dental health. Thus, the present study assessed oral and dental health findings, mean BEWE scores, and salivary cortisol levels of the elite athletes competing across different winter sports branches in Türkiye. Furthermore, the differences in salivary cortisol levels measured at 3 different times for these competitors were also compared.

Negative psychological effects due to poor oral health that cause pain and systemic inflammation adversely affect the performance of athletes.^6^ Dental caries is a worldwide health problem, which has been reported to affect 60%-90% of adults and children.^28^ A previous study conducted throughout Türkiye reported a DMFT score of 1.9 and 2.3 for 12- and 15-year-olds, respectively.^29^ Needleman et al,^6^ reported that elite athletes with a dental caries rate of 15%-75% had poor oral health, but they were similar to the people living a sedentary lifestyle in the developing countries. Yapıcı et al,^30^ in their study where they found the mean DMFT index value of male athletes was 3.9 ± 3.7, they stated that athletes with a DMFT index score below 4 performed better. Frese et al,^8^ on the other hand, could not establish any difference in the DMFT index between professional athletes and adults who did not engage in any sports discipline. In our study, the mean DMFT score was found to be 4 in the control group, while it ranged from 5 to 8.2 in the elite athletes; however, the difference was non-significant. The nutritional habits of the athletes, dehydration-associated xerostomia, insufficient oral health knowledge, and tooth brushing habits may have played a role in the participants’ higher mean of DMFT scores compared to Türkiye’s average. Oral health can be improved through simple interventions such as the use of fluoride toothpaste or a topical fluoride preparation, as well as behavioral changes related to diet, oral hygiene, and carbonated drink consumption.

There is a negative relationship between the OHI score and gingival health. Individuals with good oral hygiene have low OHI scores.^31^ Previous studies reported a plaque index score of 0.6 and 2.3 for 11-year-old competitive swimmers and professional football players, respectively.^9,32^ Our study found a clinically and statistically significant difference between the control group with good oral hygiene and elite athletes with moderate oral hygiene. Since the poor oral hygiene of the competitors is attributed to the lack of regular tooth brushing habits, instructing and practicing appropriate brushing methods are important in terms of preventing periodontal problems.

Dental injuries in athletes usually occur due to the traumas experienced during sportive activities.^27^ Dental trauma has been reported in 14%-57% of athletes in risky sports.^31^ Farcaşui et al^34^ observed that 14% of the athletes aged 6 to 13 years had experienced dental traumas and the sports disciplines most commonly associated with dental traumas were skiing and football, followed by judo. In their study, they reported that enamel fracture (66%) and uncomplicated dentin fracture (29%) were encountered most frequently.^33^ A previous study assessing the distribution of dental traumas indicated that dental traumas were mostly caused by falling and mostly observed during the winter.^35^ Considering that falling and collision risks are very high in ski jumping, ice hockey, alp discipline, and snowboarding dentoalveolar trauma cases are also most likely to occur. In the present study, dental traumas encountered in 45.1% of the athletes were mostly in the form of enamel fractures (41.8%). It was believed that the highest rate of trauma seen in alpine discipline (54.5%) was due to the fact that athletes who did not use a mouth guard took risks while competing against time, skiing aggressively and daringly under variable racetrack conditions. The lowest rate of trauma observed in ski jumping (12.5%), which is known to be a very a risky sport by the audience, might be attributed to the selection of the most suitable athletes for this discipline and avoiding trainings and competitions in dangerous weather and track conditions. To prevent dental traumas, the use of equipment such as helmets, mouth guards, and chin guards by athletes should be extended.^36^

In heavy sporting activities such as rowing and running, the prevalence of dental erosion was also reported to increase due to the high probability of gastroesophageal reflux.^37^ In the literature, the BEWE score is also used to assess erosion as well as attritions on masticatory surfaces of the teeth.^38^ A study stated that the BEWE scores of German competitive and amateur athletes between the ages of 18-30 were approximately 3.5. In our study, the highest BEWE score was 1.6 ± 1.7 in the snowboarding branch, and it is thought that the fact that this value is lower than that of German athletes is due to the fact that it was conducted on younger athletes.^39^ In the present study, the BEWE scores of the ice hockey players were similar to those of the professional ski jumpers but lower than the athletes’ in other groups. Ice hockey was the only discipline in which mouthguards are used due to the players’ physically aggressive acts and their extreme levels of physical contact associated with body-checking. It is believed that the mouthguards used by these athletes protect their teeth from attrition caused by bruxism. The extent of attrition in bruxism, which occurs in athletes as clenching or grinding teeth during the daytime or during sleep due to coach- and competition-related stress, can be exacerbated by consuming acidic sports drinks. In addition, it is believed that shivering due to cold while waiting for the race start may negatively affect the teeth, especially since alpine skiing, snowboarding, and biathlon athletes work in cold environments. The use of a mouthguard is recommended for athletes to reduce the risk of dental attrition caused by bruxism and possible tooth fractures due to traumatic injuries.

The low or high stress level or exercise intensity directly affects cortisol levels. It has been reported that cortisol levels did not increase or slightly decreased at low exercise intensity, but showed a significant increase at high intensity exercises.^40^ Moreover, there is a positive correlation between the increase in MaxVO_2_ (a measure of the maximum amount of oxygen that the body can utilize during exercise) and cortisol levels.^41^ Exercise of high intensity influences the secretory process of the adrenal cortex and starts cortisol releasing in adults and adolescents.^42,43^ Heavy training significantly increases the amount of salivary cortisol immediately after exercise. Endurance exercise produces higher plasma cortisol than acute high-intensity exercise.^44,45^ Because biathlon is a challenging combination of shooting and cross-country skiing, its athletes have a high level of long-term endurance. The fact that biathlon athletes’ cortisol levels are significantly higher than other groups at all measurement stages can be explained by their long-term endurance. The cortisol response to exercise in cold conditions is unknown. Furthermore, past studies reported that long-term adaptation to cold reduced the activity of the autonomic nervous and endocrine systems. There is, however, little evidence of the effect of either heat or cold acclimatization on the exercise-induced cortisol response.^46,47^ All measurements of our study were taken in the same environment. Additionally, the results of this study revealed that the baseline free cortisol concentrations in the elite group were consistently higher than those in the amateur group.^48^ In the present study, the cortisol level was the lowest in ski jumpers and highest in biathletes at all measurement stages. In addition, cortisol levels of biathletes were significantly higher than other groups at all measurement stages. Low cortisol levels may be linked to some circumstances that ski jumping is rather an individualized sports activity and jump ramps and outdoor conditions are also standardized. High levels of cortisol measured in the biathletes may be because the race includes rifle shooting stages and they need to exert a high level of physical force related to MaxVO_2__, _which lasts for longer periods than other athletes.

The secretion of cortisol increases due to the increase in adrenaline, and the flow of saliva released by the submandibular and sublingual glands decreases with the increase in viscosity due to the vasoconstriction of the glands through the stimulation of the sympathetic nerves. The decreased salivary flow may result in an increase in dental plaque and DMFT scores.^49^ The present study found no significant difference in DMFT scores between sports disciplines. However, it was believed that the significantly higher OHI-S score of the Biathlon group, which had the highest cortisol level among the other sports disciplines was caused by the decreased salivary flow due to the increased stress and adrenaline levels in this discipline. Additionally, an increased level of cortisol, indicating an increase in stress levels, is clinically associated with bruxism. And in the long term, bruxism can lead to tooth mobility and tooth loss.

It may be more enlightening to plan new scientific studies investigating the effects of individual and team winter sports and the impacts of standard or variable environments where they are played on stress levels of their competitors. To reduce the negative health effects of stress, it may be beneficial to perform the trainings in a race format. It would be beneficial to conduct more studies to confirm the potential relationships about the effect of sporting activities on oral-dental health.

Scarcity of dental trauma findings in winter sports athletes who commonly fall indicates that these sports branches do not have any dental trauma risk. However, it was determined that the oral hygiene level in the winter sports branches was lower than the control group. Elite athletes are expected to have good general health and to perform at their best in competitions. Nonetheless, it is important that they have optimum oral health and pay attention to the preventive oral care so that dental problems do not adversely affect their success. In addition, the stress levels measured in the athletes of ski jumping, known as an adrenaline sport, was found to be lower in the control group, contrary to the expectations, whereas higher level of stress had been observed in the biathlon group. Bruxism, which is the intraoral reflection of stress, was found to be lower in the hockey group than the control group. The high stress levels in winter sport branches underline the need for stress control methods and the use of a mouth guard in competitive sports. In addition to performing periodic oral and dental examinations, stress control methods should also be incorporated into the preventive health care of elite athletes to ensure that all athletes are in good health conditions during the competitions.

### Perspective

Among all sports branches, the lowest dental attrition scores seen in the ice hockey players are supporting the efficacy of the mouth guards. Post-competition salivary stress biomarker levels of the athletes were found to be higher than the pre-competition and during-match stress levels. Cortisol levels, measured in saliva, may promote the optimization of athletes’ training and match standards.

## Figures and Tables

**Table 1.** Distribution of Athletes According to Winter Sports Branches and the Average Age

Branch	N	Age	
		Mean ± Standard Deviation	Min-Max
Snowboard	11	14.1 ± 0.8^c^	12.9-15.3
Ice hockey	22	15.1 ± 0.4^a^	13.6-15.8
Alpine skiing	11	14.2 ± 1.0^b^	12.8-15.6
Ski jumping	16	13.5 ± 1.1^c^	12.2-15.1
Biathlon	11	14.8 ± 0.6^ab^	13.6-15.8
Control group	20	15 ± 0.7^ab^	13.3-16.6
*P*		<.001	

There was no statistically significant difference between groups with common letters.

**Table 2.** Distribution of DMFT, DMFS, PUFA, and Oral Hygiene Index Scores of Different Winter Sports Branches

Branch	DMFT		DMFS		PUFA>0		OHI-S	
	Mean ± SD	Min-Max	Mean ± SD	Min-Max	n	%	Mean ± SD	Min-Max
Snowboard	5.0 ± 3.1	0-11.0	7.7 ± 5.3	0-18.0	2	18.2	1.3 ± 0.4^b^	0.3-2.3
Ice hockey	5.9 ± 2.8	1-11.0	8.7 ± 5.9	1-28.0	6	27.3	1.4 ± 0.5^ab^	0.5-2.5
Alpine skiing	8.2 ± 4.3	2-17.0	14.1 ±7.6	2-26.0	4	36.4	1.5 ± 0.2^ab^	1.1-2.0
Ski jumping	5.3 ± 3.0	0-11.0	8.8 ± 6.4	0-24.0	0	0	1.5 ± 0.7^ab^	0.3-2.8
Biathlon	7.6 ± 3.6	3-17.0	10.8 ± 4.8	3-21.0	0	0	1.7 ± 0.4^a^	0.8-2.6
Control group	4.0 ± 3.0	0-9.0	7.0 ± 6.4	0-19.0	1	5	1.1 ± 0.4^c^	0.3-2.0
*P*	.083		.077				.018*	

SD, standard deviation.

*There is a significant difference as a result of the Kruskal–Wallis analysis (*P *< .05).

**Table 3.** Distribution of Basic Erosive Wear Examination Scores of Athletes

Branch	Total BEWE Score	
	Mean ± SD	Minimum–Maximum
Snowboard	1.6 ± 1.7^a^	0-6.0
Ice hockey	0.4 ± 0.6^b^	0-2.0
Alpine skiing	1.2 ± 0.9^a^	0-2.0
Ski jumping	1 ± 1.0^ab^	0-3.0
Biathlon	1.4 ± 1.1^a^	0-3.0
Control group	1.3 ± 1.5^a^	0-6.0
*P*	.034*	

BEWE, basic erosive wear examination; SD, standard deviation.

*There is a significant difference in the result of the Kruskal–Wallis analysis (*P* < .05).

**Table 4.** Salivary Cortisol Level Mean and Standard Deviations of Athletes in Different Branches Before, During, and After the Race (Mean ± SD)

Branch	Salivary Cortisol Levels						
	Pre-Competition		During Match		Post-Competition		*P*
	**Mean ± SS**	Minimum–Maximum	Mean ± SS	Minimum–Maximum	Mean ± SS	Minimum–Maximum	
Snowboard	276 ± 147^bc^	110-573	340 ± 232^b^	29-694	445 ± 195^b^	175-784	.18
Ice hockey	252 ± 148^bc^	17-530	279 ± 169^bc^	17-673	381 ± 329^b^	19-1096	.186
Alpine skiing	221 ± 158^c,x^	80-597	311 ± 123^b,xy^	155-548	401 ± 237^b,y^	137-870	.029**
Ski jumping	165 ± 177^c^	12-508	172 ± 127^c^	11-361	181 ± 136^c^	11-407	.779
Biathlon	637 ± 140^a^	434-847	615 ± 144^a^	449-856	615 ± 142^a^	445-852	.695
Control group	311 ± 79^b^	140-459					
Elite athletes	291 ± 216^x^	12-847	321 ± 207^x^	11-856	385 ± 267^y^	12-1096	<.05***
*P*	<.001*		<.001*		<.001*		

*There is a significant difference in the result of the Kruskal–Wallis analysis (*P* < .05)

**There is a significant difference in the result of Friedman’s analysis (*P* < .05).

***There is a significant difference in the results of the *t*-test in paired groups (*P* < .05).

^a,b,c^There is no statistically significant difference between groups with common letters in the same column.

^x,y^There is no statistically significant difference between groups with common letters on the same line.
